# Role and relevance of dentists in a multiprofessional palliative care team: results of a cross-sectional survey study

**DOI:** 10.1007/s00520-024-08356-y

**Published:** 2024-02-15

**Authors:** Greta Antonia Weihermann, Florian Bernhardt, Tobias J. Brix, Sebastian-Edgar Baumeister, Philipp Lenz

**Affiliations:** 1 Department of Palliative Care, University Hospital Muenster, 48149 Muenster, Germany; 2 Institute of Medical Informatics, University of Muenster, 48149 Muenster, Germany; 3 Institute of Health Services Research in Dentistry, University of Muenster, 48149 Muenster, Germany; 4 Department of Palliative Care, University Hospital Muenster, Albert-Schweitzer-Campus 1, Building W 30, D-48149 Muenster, Germany

**Keywords:** Palliative care, Palliative medicine, Supportive care, Dentistry, Advanced cancer

## Abstract

**Purpose:**

Despite the multiprofessional concept surrounding palliative care patients (PCPs) and their high prevalence of oral issues, licensed dentists (LDs) are often not included in their treatment team. This study aimed to examine the current state of cooperation and to determine whether and how LDs should be included in the care for PCPs.

**Methods:**

This single-centre cross-sectional study was conducted at the University Hospital Muenster, Germany. We surveyed three participant groups: PCPs, LDs, and healthcare professionals (HCPs). Questionnaires were tailored for each group, with some questions common for comparison.

**Results:**

The study encompassed the results of 48 questionnaires from LDs, 50 from PCPs along with 50 from HCPs. Consensus was reached among all parties (LDs: 73% (*n* = 35/48); HCPs: 94%, *n* = 47/50; PCPs: 60%, *n* = 30/50) that involving LDs in the treatment concept is favourable. On the other hand, a significant discrepancy emerged in the perception of the dental treatment effort required by PCPs. While LDs (81%; *n* = 39/48) and HCPs (64%; *n* = 32/50) were convinced of increased effort, PCPs (34%; *n* = 17/50) largely did not share this perspective. To enhance patient care and formulate appropriate treatment plans, LDs consider both training (58%; *n* = 28/48) and guidebooks (71%; *n* = 34/48) to be valuable and would attend or use such resources.

**Conclusion:**

This study sheds light on the current gaps in including LDs in palliative care teams and emphasizes the importance of multidisciplinary collaboration to address oral health needs effectively. Development of continuing education options and collaborative models between LDs and HCPs needs to be further expanded in future.

## Introduction

The fact that the world’s population is aging at an increasingly faster rate each year, resulting in a shift in the age pyramid towards the top, is not new. According to WHO projections, the proportion of people aged 60 and over will nearly double from 12 to 22% between 2015 and 2050. The proportion of people aged 80 and over will even triple between 2020 and 2050. One accompanying consequence is the increasing incidence of cancer [1] or complex multimorbidity [2]. In 2020, an estimated 19.3 million new cancer cases and nearly 10 million cancer-related deaths were recorded. By 2040, the global cancer burden is expected to increase by 47% to about 28.4 million cases [3]. This, among other factors, accounts for the ever-increasing need for palliative care (PC) worldwide. Nevertheless, it was reported in only 40% of all countries that PC is available in principle. It should be noted that mainly countries in the European region and high-income groups (over two-thirds) have and utilize such services, whereas it is less common in the low-income group [4]. Moreover, PC is often misinterpreted as solely end-of-life care and is thus often initiated too late [5], although interdisciplinary teams can effectively address the care needs of patients to reduce end-of-life crises [6].

Also, an increasing complexity of care needs is recognized [7]. Therefore, improvements in structure and education for PC seems necessary [7–9]. This requires guidance structures that can indicate when the ideal starting point for treatment is, which procedures can be used for which patients and to what extent [6], and which health care professionals (HCPs) should be involved.

Despite the multidisciplinary philosophy of PC, a licensed dentist (LD) is often not part of the team, although many patients suffer from oral problems either caused by (cancer) therapy or by the oral disease itself [10]. These symptoms in the oral cavity (e.g., ulcers, mucositis, pulpitis, and abscesses) have a negative impact on overall health and quality of life [11]. Cancer patients often complain about dry mouth, Candidiasis infection, and loss of masticatory function [10]. Dental-supported PC should, as far as possible, maintain optimal function, reduce the microbial burden in the mouth and, thus, the risk of pain and oral risks, and ultimately contribute to the overall goal of increased quality of life [12].

The lack of appropriate inclusion of dental care in the treatment of PC patients seems to be not solely because “traditional” PC teams are not aware of the importance of dental care. According to a study by van der Valk et al., neither dental students nor LDs show a particular affinity for palliative patients, nor do they seek to be involved in the treatment processes. However, they are well aware of the potential benefits for palliative patients in terms of oral health and quality of life by including LDs [13].

Thus, the present study sought to assess the existing level of cooperation and ascertain if and how LDs should be integrated into the care for palliative care patients (PCPs).

## Material and methods

### Study design

This cross-sectional study was performed among three participant groups at the University Hospital Muenster (UKM), Germany. The study protocol conformed to the ethical guidelines of the Declaration of Helsinki and was a priori approved by the local ethics committee of the University of Muenster (2022–125-f-S). Recruitment began in Mai 2022 and ended in November 2022. We used the STROBE checklist for cross-sectional studies checklists when writing our report [14].

In this study, PCPs, LDs, and HCPs involved in PC were anonymously surveyed regarding dental health topics. The participants completed the questionnaires either on a tablet or using an online survey tool, which took approximately five to ten minutes depending on the group being surveyed. The three different participant groups each received different tailored, yet unvalidated questionnaires. These inquiries often revolved around the extent of an interdisciplinary setup of palliative teams, reasons for the lack of LDs involvement, and optimal approaches for designing future solutions. To ensure comparability in certain aspects, some questions were present in all three questionnaires. In terms of questionnaire-design, options for multiple-choice, ranking, text fields for input and Likert scales were utilized. A 6-point Likert scale was used in the questionnaire for the HCPs, aiming to assess how important the participation of LDs within the PC team was as perceived by the HCPs. Here, 1 signified "very important" and 6 indicated "completely irrelevant".

### Setting and participants

Our sample included 150 contacted LDs, of which ultimately 60 (40%; 60/150) records were created. Thereof, 48 LDs (80%; 48/60) agreed to participate and completed the survey, while 4 LDs (7%; 4/60) agreed to participate but discontinued data entry during the process. Besides, 4 LDs (7%; 4/60) consented to participate but subsequently did not answer any questions. Also 4 (7%; 4/16) LDs did not grant consent, hence the questionnaire remained uncompleted. Out of 58 data sets of PCPs, 50 (86%; *n* = 50/58) of them granted their consent and were subsequently interviewed. In the group of PCPs, 7 (12%; *n* = 7/58) consented to participation, of whom 2 (3%; *n* = 2/58) only partially completed the questionnaire, while 5 (9%; *n* = 5/58) subsequently did not answer a single question. One PCP (2%; *n* = 1/58) did not grant consent, and the survey was terminated at that point. Out of the HCPs, 50 (94%; *n* = 50/53) consented and were subsequently surveyed, while 3 (6%; *n* = 3/53) participants consented but did not answer a single question thereafter. The delineated results exclusively concern the datasets of participants who diligently completed the questionnaire in its entirety. Should individual inquiries have remained unanswered, they were duly noted under the category of 'not specified' and likewise represented in the graphical representations. Similarly, in the calculations of percentages, these instances were incorporated into the overall count, thereby contributing to the reference base. We have deliberately opted to include and present any missing responses in all relevant figures as this information may provide valuable information about the validity of the questions posed. Those respondents who did not complete the questionnaire in its entirety, and those who did not provide their consent, were excluded from the data analysis. In the case of conditional queries, the reference bases for the respective percentage values were exclusively comprised of respondents to whom the previously inquired conditions applied. Since the survey was intended to be completely anonymous, we deliberately refrained from collecting any information on gender, age, or ethnicity of the LDs and HCPs. All three groups of respondents received their respective questionnaires via a handheld tablet (HCPs and PCPs), where the questionnaire could be accessed, or through an internet link (LDs) leading to the online questionnaire. Prior to the actual questionnaire opening, participants from all three groups were required to provide their consent to participate in the study. Study data were collected and managed using REDCap electronic data capture tools. REDCap (Research Electronic Data Capture) is a secure, web-based software platform designed to support data capture for research studies, providing 1) an intuitive interface for validated data capture; 2) audit trails for tracking data manipulation and export procedures; 3) automated export procedures for seamless data downloads to common statistical packages; and 4) procedures for data integration and interoperability with external sources [15, 16]. The PCPs were admitted to the UKM for inpatient care, receiving oncological treatment and/or PC. In addition, certain descriptive data on the PCPs and their general treatment were pseudonymously obtained through the in-house electronic hospital information system (ORBIS by Dedalus). PCPs were interviewed in their respective rooms where they were accommodated. Those who were unable to fill out the questionnaire independently or read the questions received neutral assistance on-site by the first author. The participants of the HCP group were all employed at the UKM. They were either gathered for the survey during the hospital's regular activities or interviewed during free periods directly on the ward. The final participant group consisted of LDs who were randomly selected from a list of all LDs of the state of North Rhine-Westphalia, Germany, and contacted by email or telephone. They were directed to the online questionnaire through a link in the received email.

### Statistics

Descriptive statistics were used to analyze demographic data. We summarized continuous variables mainly by the mean and standard deviation (SD). Categorical variables are presented as absolute and relative frequencies. Statistical analyses were performed using SPSS Software (IBM Corp. Released 2017. IBM SPSS Statistics for Mac, Version 25.0. Armonk, NY: IBM Corp.) and SAS Software (Version 9.4, SAS Institute Inc., Cary, NC, USA). In all analyses, percentage values were rounded to whole numbers due to limited sample sizes per group for better clarity. For enhanced clarity, the absolute numbers for the number of participants who answered the specified questions are always provided.

## Results

The present study is based on 48 questionnaires from LDs, 50 from PCPs along with 50 from HCPs. Table 1 indicates how long ago the participating LDs obtained their license, specifies professional categories to which the surveyed HCPs belong and gives basic characteristics regarding PCPs.

**Table 1:** Composition of the three participant groups

Time since approval of dental license for LDs (*n* = 48)		
Within the last 5 years; *n* (%)		3 (6%)
5–10 years ago; *n* (%)		3 (6%)
10–15 years ago; *n* (%)		8 (17%)
More than 15 years ago; *n* (%)		34 (71%)
Composition of HCPs (*n* = 50)		
Oncology nurses; *n* (%)		29 (59%)
Physiotherapists; *n* (%)		12 (25%)
Primarily non-oncology nurses; *n* (%)		3 (6%)
Primarily non-oncology doctors; *n* (%)		3 (6%)
Primarily oncology doctors; *n* (%)		2 (4%)
Not specified		1 (2%)
Characteristics of PCPs (*n* = 50)		
Sex	Female; *n* (%)	28 (56%)
	Male; *n* (%)	22 (44%)
Age (years)	Mean	66.6
	Min	28
	Max	93
	SD	14.109
Days of treatment	Mean	34.62
	Min	4
	Max	90
	SD	20.949
	Median	31.5
	IQR	29
Type of diagnosis	Malignant; *n* (%)	39 (78%)
	Benign; *n* (%)	8 (16%)
	Hematologic / Lymphatic; *n* (%)	3 (6%)
Tumor location affected by the condition	Gynecological; *n* (%)	9 (18%)
	Lungs; *n* (%)	8 (16%)
	Nervous system; *n* (%)	5 (10%)
	Gastrointestinal tract; *n* (%)	4 (8%)
	Multiple malignancies; *n* (%)	4 (8%)
	Ear, Nose, and Throat (ENT); *n* (%)	3 (6%)
	Urinary tract; *n* (%)	3 (6%)
	Orthopedics; *n* (%)	2 (4%)
	Skin; *n* (%)	1 (2%)

Abbreviations: *LDs* licensed dentists, *HCPs* health care professionals, *PCPs* palliative care patients, *Min* Minimum, *Max* Maximum, *SD* Standard deviation, *IQR* Interquartile range

### Licensed dentists

Of the LDs, 50% (*n* = 24/48) indicated that they already treated PCPs, 23% (*n* = 11/48) never cared for PCPs but thought, that they would be able to, and 25% (*n* = 12/48) preferred to refer these patients directly. The latter group consisted of 83% (*n* = 10/12) of LDs whose license was more than 15 years old.

**Fig. 1:**
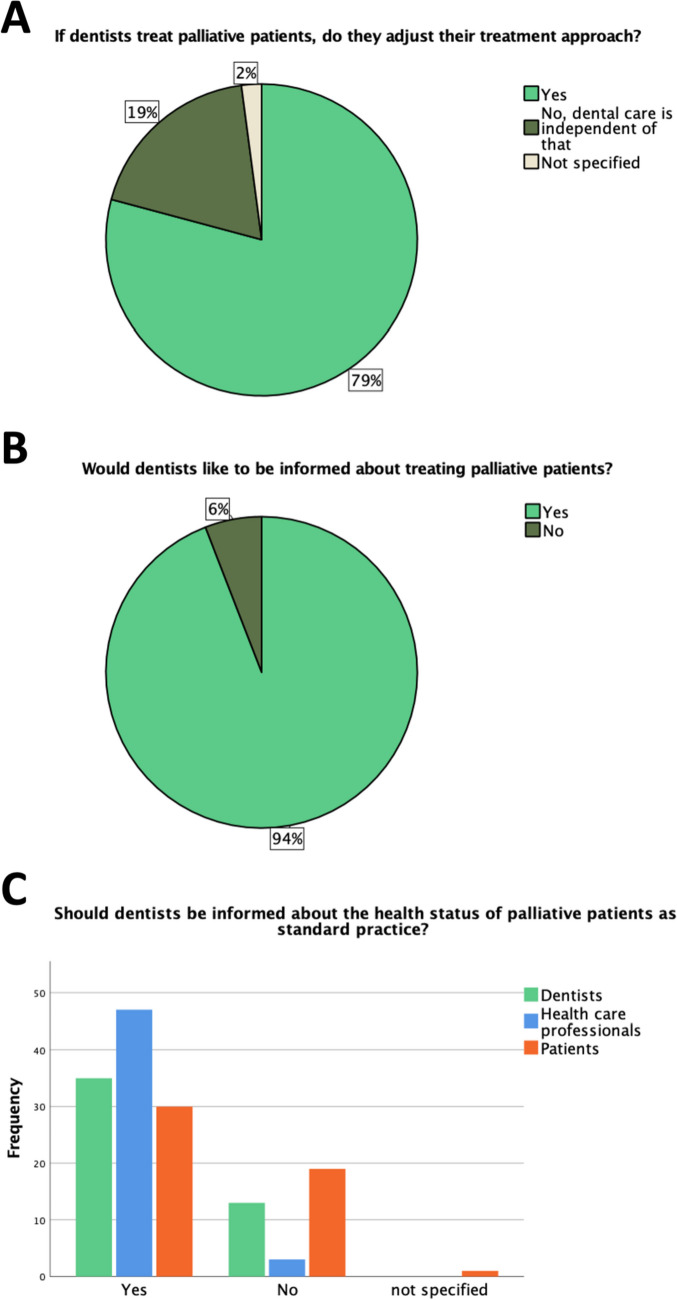
Graphical representations of the questions.** A** “If dentists treat palliative patients do they adjust their treatment approach?” (*n* = 48) **B** “Would dentists like to be informed about treating palliative patients?” (*n* = 48) **C** “Should dentists be informed about the health status of palliative patients as standard practice?”; Statements divided for 3 participant groups (licensed dentists: *n* = 48; health care professionals: *n* = 50; palliative care patients: *n* = 50); not specified = the respondent skipped the question or did not select any answer option

As shown in Fig. 1, most of the LDs indicated that they would adjust their treatment concept according to the patient (79%; *n* = 38/48). Of these 38 LDs, 32 (84%) provided a more detailed statement: In particular, it was often mentioned that the primary goal of treatment is pain relief (31%; *n* = 10/32). Furthermore, LDs described several times that only necessary measures are carried out which are tailored to the patient's capacity to endure in relation to their disease, general condition, and life expectancy (56%; *n* = 18/32). Moreover, they emphasized that the treatment should be adapted to the patient's quality of life and expectations (28%; *n* = 9/32). The LDs who provide care to PCPs have been predominantly informed about their health condition by PCPs themselves (46%, *n* = 11/24), followed by the patients' relatives (29%, *n* = 7/24). In general, nearly all LDs (Fig. 1; 94%; *n* = 45/48) wanted to be informed if they are caring for PCPs. If the LD’s notification would be mandatory, the most preferred means of information among LDs was a personal conversation with PCPs (29%; *n* = 14/48). Due to the demographic shift, LDs expect that there will be more visits from PCPs in the future (73%; *n* = 35/48). However, 75% (*n* = 36/48) also thought that these patients will continue to be referred to clinics, as knowledge about the treatment of PCPs is unlikely to improve.

**Fig. 2:**
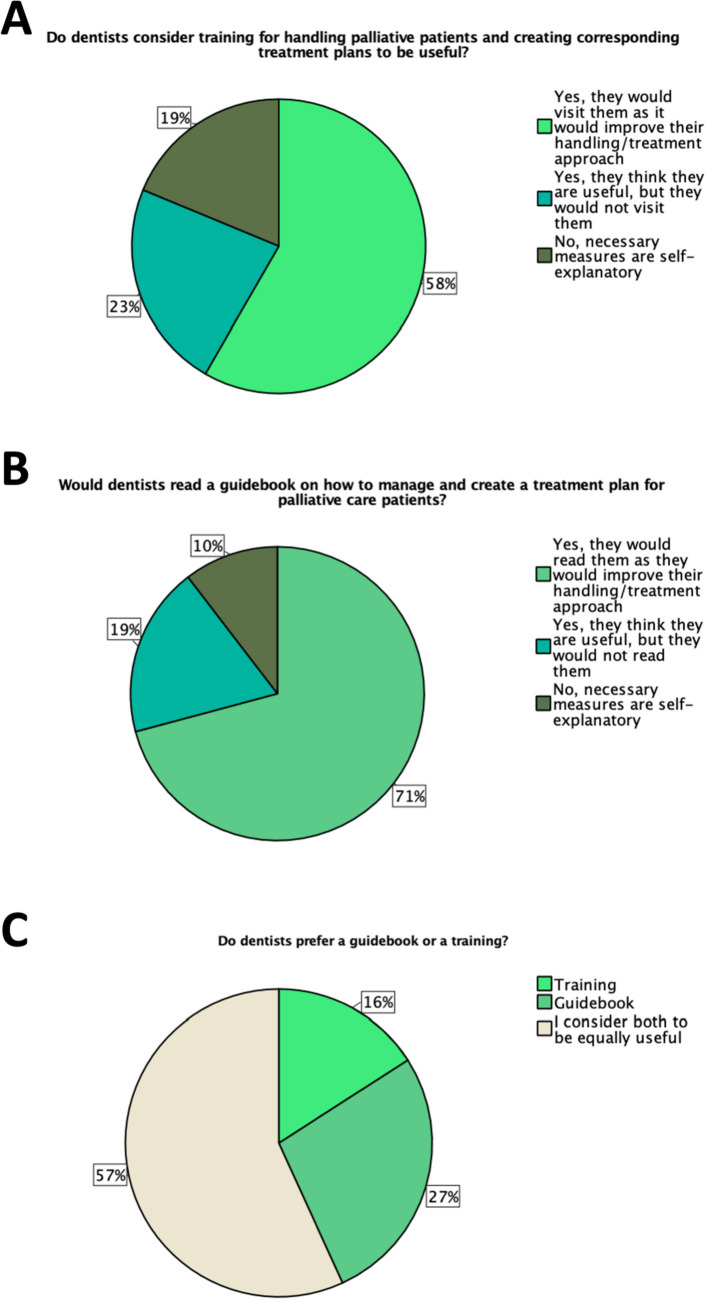
Pie charts for the questions.** A “**Would dentists consider training or **B** read a guidebook for handling palliative patients and creating corresponding treatment plans to be useful?”; (*n* = 48) **C** “Do dentists prefer a guidebook or a training?” (*n* = 44)

If training were offered to improve the handling of PCPs, more than half of the surveyed LDs (58%; *n* = 28/48) claimed that they would attend and considered it useful. Even better than training is the assessment of a continuing education guidebook, which more than 70% (71%; *n* = 34/48) of the LDs considered useful and would read. Yet, 57% (*n* = 25/44) of LDs consider both options to be equally valid. See Fig. 2 for more detailed information on this topic.

**Fig. 3:**
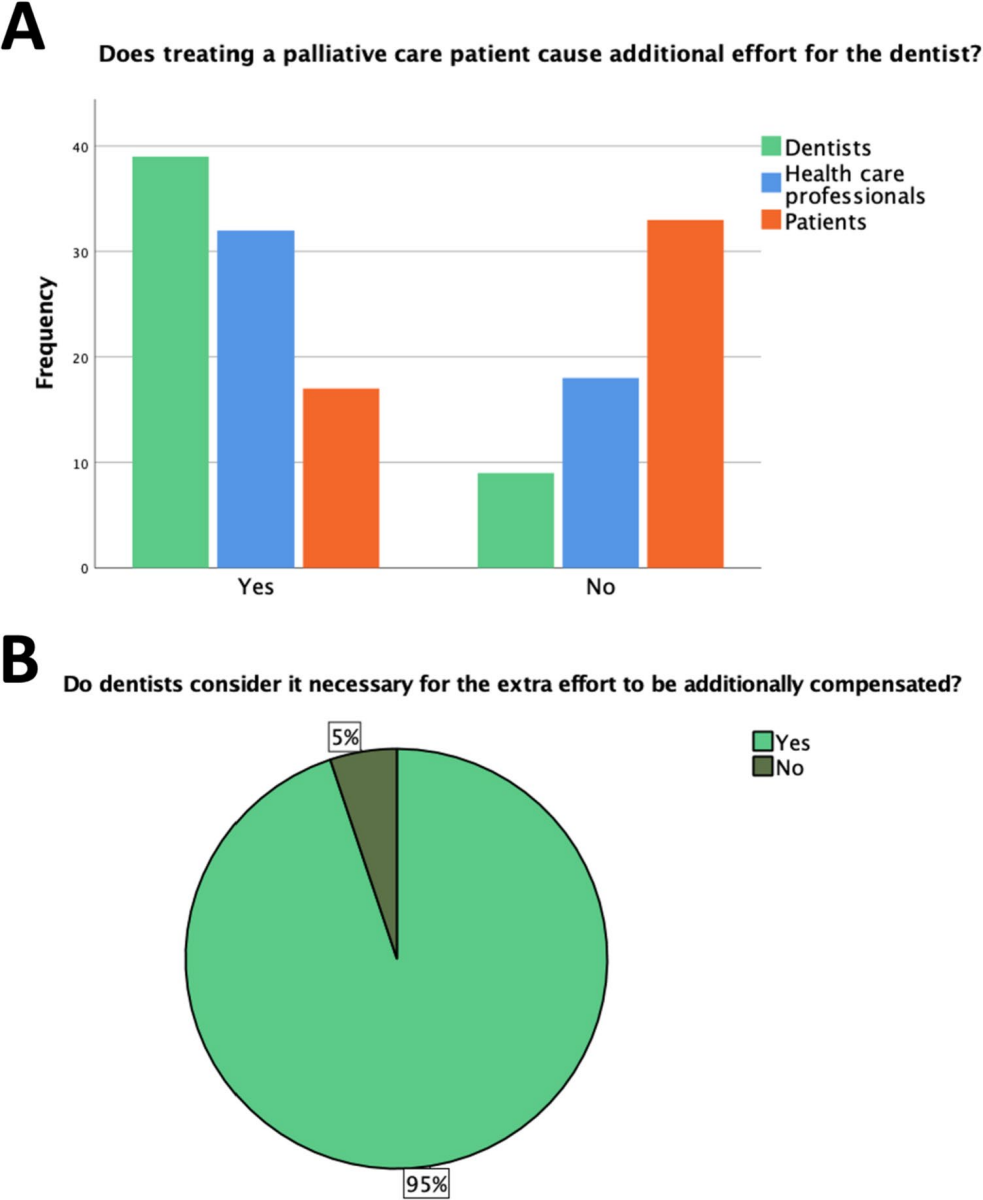
Graphical representations of the questions.** A “**Does treating a palliative care patient cause additional effort for the dentist?”; statements divided for 3 participant groups (licensed dentists: *n* = 48; health care professionals: *n* = 50; palliative care patients: *n* = 50) **B** “Do dentists consider it necessary for the extra effort to be additionally compensated?” (*n* = 39)

As illustrated in Fig. 1, nearly three-quarters (73%; *n* = 35/48) of the LDs believed that they should be informed about the health status of patients with a progressive incurable disease. However, the majority of LDs (81%; *n* = 39/48) believed that treating a PCP is an additional effort, of which 95% (*n* = 37/39) would like to be financially compensated (Fig. 3).

### Palliative care patients

PCPs themselves assessed this question differently, with only less than a half (34%; *n* = 17/50) assuming that they do represent an additional burden for the dentist in their treatment, as shown in Fig. 3. Moreover, dental care in general was very important to the majority: Only 12% (*n* = 6/50) of patients for whom this was always important said that it has not been important since they received their diagnosis. The majority of PCPs (72%; *n* = 36/50) relied on their LD´s opinion and would always act according to their advice regarding dental findings requiring treatment. Furthermore, a large proportion would also like to invest in dental services (88%; *n* = 44/50). Therefore, more than 60% (*n* = 30/50) considered it important that the LD is involved in the general treatment process (Fig. 1). As shown in Table 2, PCPs do indeed experience issues in the oral area that fall within the scope of a LD´s responsibilities. Only 6/50 (12%) PCPs indicated that they had no issues in the oral cavity.

**Table 2:** Oral issues of the surveyed palliative care patients (*n* = 50)

Oral issues of the surveyed palliative care patients (*n* = 50)	
Xerostomia; *n* (%)	28 (56%)
Aesthetics; *n* (%)	23 (46%)
Mucositis and gingivitis; *n* (%)	20 (40%)
Ability for independent chewing; *n* (%)	18 (36%)
Speaking; *n* (%)	17 (34%)
Disturbances in taste; *n* (%)	17 (34%)
Pain while swallowing / during food intake; *n* (%)	15 (30%)
Canker sores; *n* (%)	12 (24%)
Halitosis; *n* (%)	10 (20%)
Pharyngitis; *n* (%)	5 (10%)
Persistent chronic pain sensation in the mouth; *n* (%)	5 (10%)
No impairments in the oral area; *n* (%)	6 (12%)

**Fig. 4:**
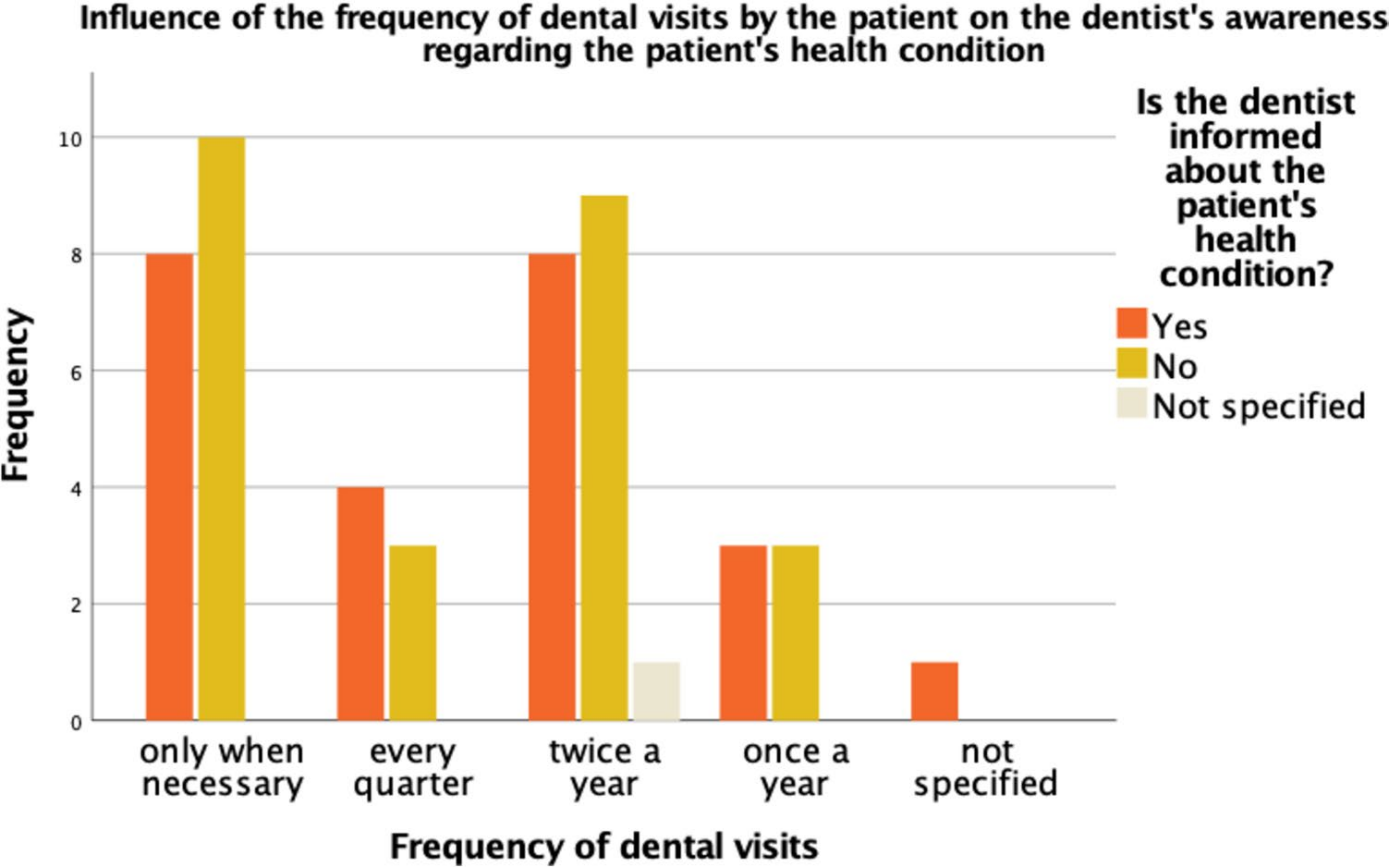
Bar chart representing the influence of the frequency of dental visits by the patient on the dentists’ awareness regarding the patient`s health condition; divided by frequency of dental visits (*n* = 50); not specified = the respondent skipped the question or did not select any answer option

However, regardless of how often PCPs visited their LDs, only about half of LDs (48%; *n* = 24/50) were informed about their patients' overall health status (see Fig. 4). If LDs have been notified, according to the PCPs, in 88% (*n* = 21/24) of these cases information transfer was carried out by themselves. If the notification of the LDs were mandatory, patients preferred a personal conversation (52%; *n* = 26/50). Of the PCPs where LDs were not involved, 68% (*n* = 17/25) stated that informing the LDs was not necessary.

When asked about communication between the treating medical team and the LDs, 80% (*n* = 40/50) of PCPs reported that no information exchange had ever taken place.

### Health care professionals

Of the HCPs, 66% (*n* = 33/50) reported that most of their patients do have oral health problems that are burdensome to them. In addition, nearly all of the HCPs (94%; *n* = 47/50) believed that a LD should be informed by the PC team about the health status of affected PCPs as a standard procedure (Fig. 1). The significance of a dentist's participation within a PC team was assessed by HCPs on a Likert scale from 1 to 6, with 94% rating it between 1 and 3 (*n* = 47/50) (median: 2.0).

Regarding the transfer of information, HCPs considered notifying the LDs about the health status of their patients via PC physicians and phone calls (40%; *n* = 20/50) to be the best option.

Concerning whether PCPs mean an additional workload for LDs, 64% (*n* = 32/50) of the HCPs answered positively, lying between the estimates of LDs and PCPs (Fig. 3).

## Discussion

According to our results, HCPs are indeed aware of the problems their patients experience in the oral cavity, with dry mouth, aesthetics, and mucositis or gingivitis playing crucial roles, according to our surveyed patients. The lack of interventions could be explained by the fact that, for example, oncologists face great difficulties in treating oral problems [17] since dental education and care are traditionally separated from medical care. Consequently, neither physicians nor HCPs are sensitized to oral problems in end-of-life care [18]. However, LDs are not routinely involved in PC, which is supported by not only our results but also numerous other studies [18–21]. One study even showed that the oral health of terminally ill cancer patients deteriorated under PC, despite receiving oral care from nurses [22]. However, a trained LD may help other physicians deal with these situations [17] and thus could complete the multiprofessional treatment philosophy of PC effectively.

However, one key question is how the treating HCPs, LDs, and ultimately the PCPs may better come together. Especially in context of the increasing number of individuals receiving specialized PC in inpatient and outpatient settings, the integration of diverse disciplines is essential yet difficult to ensure on-site on short term. From this, it can be inferred that information exchange among various disciplines is an indispensable prerequisite for multidisciplinary collaboration. This raises the question of how digital technologies may be employed for this purpose. For example, our own working group recently highlighted that the utilization of telemedical consultations significantly simplifies and even enables the short-term involvement of other specialized disciplines in PC [23]. Telemedicine describes medical practices conducted remotely and has notably taken a significant leap forward, especially during the COVID-19 pandemic [24]. Consequently, today, not only can patients themselves benefit from this by avoiding unnecessary referrals and transfers, but also attending physicians, who respond to this concept with a high level of satisfaction [23]. However, the balance between technology and personal care still needs to be perfected [25]. Although LDs may have limitations in remote diagnosis compared to standard diagnostic procedures such as palpation or auscultation. Studies have shown that the use of telemedicine programs has contributed to reducing costs, waiting times, and unnecessary referrals. In this context, it has been demonstrated that teledentistry is primarily utilized by patients who are either physically restricted or unable to travel for various reasons [26]. Though, why don't HCPs use telemedicine to assess whether a referral to the appropriate specialty and, if necessary, a physical visit of a patient could be necessary and beneficial? For example, if a patient is receiving PC and complains of oral issues, the attending PC physician could contact a LD through telecommunication and, if necessary, jointly decide on the need for a dental visit (possibly with the help of relevant photos or videos). Yet, implementing such concepts would benefit strongly from appropriate guidelines and protocols, which unfortunately do not currently exist.

Also, does PCPs currently know that it is important for LDs to be informed about their status being palliative? This might not initially seem relevant to the oral cavity at first sight. As most patients are not likely to recognize this, it may be advisable to reconsider the involvement of HCPs at this juncture. Their broad medical knowledge may enable them to at least assess the potential issues that may arise or have already occurred due to medications and, therefore, warrant further evaluation by LDs. Interestingly, in our study, only HCPs indicated that it would be beneficial for LDs to be informed by PC staff.

Furthermore, patients often do not know exactly what prognosis they have. Even cancer patients who claim to have been informed about their prognosis by their doctors only have a no or a limited understanding of the explanations provided by their treating physicans [27]. Therefore, it should be even more important for LDs to have access to the patient's medical data to tailor the treatment ideally to the patient and their specific needs. One possible solution could be electronic health records, which have mostly been used as a test design and focused on emergency medicine. These should include a brief summary of the patient's medical history, along with a list of important medications and allergies. Many studies and medical staff agree that this may be very useful and, consequently, improve patient treatment [28–30]. And isn't that ultimately the crucial point: That the patient benefits from the best possible treatment for their health? Thus, this may potentially serve as a "medical network” through the establishment of”interprofessional relationships" [28].

Therefore, we believe that LDs should be included in this medical network for PC patients in the future, when patients are in need of oral treatments. Both from the providers' as well as the patients' point of view, this would entail great advantages given the high rates of oral issues, which should not be neglected.

## Limitations

In order to contextualize our findings, it is essential to bear in mind that the number of study participants, each comprising around 50 individuals, is relatively limited. In addition, it should also be noted that participants from both the HCP group and the PCP group all worked or received treatment at the same hospital. Similarly, the LDs all came from a limited area in North Rhine-Westphalia. Therefore, it is important to recognize that the results may not necessarily reflect the situation in other regions of Germany or globally. Furthermore, it should be mentioned that all three questionnaires used were developed based on questions raised in current publications with relevant content. Yet, these questionnaires were only created for this study and have not been tested or validated before.

## Conclusion

As not only our findings, but also other studies indicate, HCPs and LDs are well aware that PCPs often encounter oral health issues. However, in the majority of cases, dental and palliative healthcare are completely disconnected from each other at present. To enable more seamless integration of LDs into a multidisciplinary PC team, it would be beneficial if LDs were provided with basic medical information about PCPs. Subsequently, this could enable focused communication with the primarily attending PC staff. To familiarize LDs with treatment and planning for PCPs, the introduction of currently absent guidebooks or training programs might also be advisable. According to our findings, both resources would likely be well-received and effectively utilized by LDs.

## Data Availability

The datasets used and/or analyzed during the current study are available from the corresponding author on reasonable request.

## Abbreviations

HCPs: Health care professionals
LDs: Licensed dentists
PC: Palliative care
PCPs: Palliative care patients
SD: Standard deviation
